# Fluorescence Imaging in Genipin Crosslinked Chitosan–Poly(vinyl pyrrolidone) Hydrogels

**DOI:** 10.3390/polym8110385

**Published:** 2016-10-28

**Authors:** Simon Matcham, Katarina Novakovic

**Affiliations:** School of Chemical Engineering and Advanced Materials, Newcastle University, Newcastle upon Tyne NE1 7RU, UK; simon.matcham@hotmail.co.uk

**Keywords:** genipin, chitosan, smart hydrogel, fluorescence, pH, temperature

## Abstract

Recent research has identified genipin as a promising natural crosslinking agent for biocompatible hydrogels as genipin is significantly less cytotoxic than current synthetic crosslinking agents, such as glutaraldehyde. Conveniently, fluorophores can be produced when genipin crosslinks. In this study, fluorescence intensity measurements of genipin crosslinked chitosan-poly(vinyl pyrrolidone) hydrogels have been explored as a dynamic, in situ method for tracing sol-gel transition. These pH-responsive smart materials have a future in medical applications, in particular in tissue engineering and drug delivery, where methods to follow the process in situ and in real-time are crucial for future advancement. Samples were prepared using deionised water, pH 4, and pH 10 solutions, and studied at 24 and 37 °C over a 24 h period. Both temperature and pH have been found to affect sol-gel transition in the hydrogels studied. The transition from acidic (pH 4) to basic (pH 10) solution resulted in reduced fluorescence intensity suggesting that, under more basic conditions, genipin molecules self-polymerise, reducing the number of molecules available for reaction with the amino groups of chitosan. Three-dimensional representations of the fluorescence present in a hydrogel sample have also been produced from the data, enabling the visualisation of variation in fluorescence with time at the surface of the hydrogel.

## 1. Introduction

**Figure 1:**
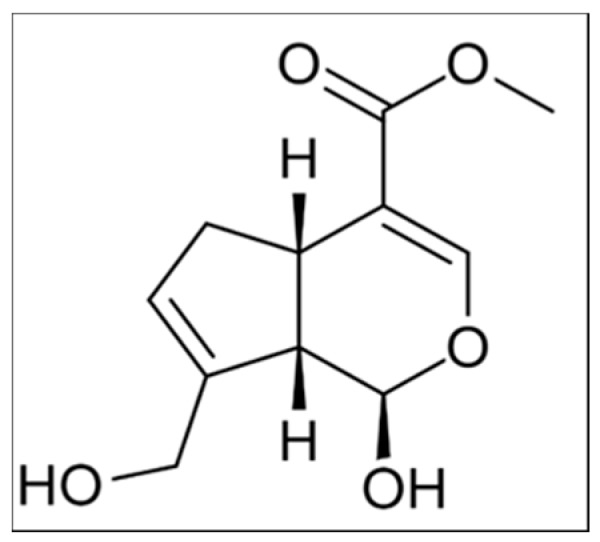
Genipin.

Hydrogels are three-dimensional macromolecular networks of crosslinked polymer chains which are capable of retaining large amounts of water due to the hydrophilic functional groups which they contain. This makes them ideal candidates for applications such as drug delivery, wound dressings, and tissue engineering, due to their similarity to organic tissue. An example of a promising hydrogel is genipin-crosslinked chitosan-poly(vinyl pyrrolidone). Chitosan is a linear, natural polysaccharide derived from the deacetylation of chitin obtained from crustacean and fungal mycelia [1]. Structurally, it mimics glycosaminoglycans, an important component of the extracellular matrix in many tissues and organs. Chitosan has been widely studied and recognised as a promising pH-responsive biomedical material [2]. The addition of poly(vinyl pyrrolidone) (PVP) enhances moisture penetration and improves dispersion, allowing for enhanced matrix formation. Polymer chains of PVP are trapped within the polymer network. This creates a semi-interpenetrating polymer network, where a network is partially intertwined with polymer chains which are not covalently bonded to it, but cannot be easily separated unless chemical bonds are broken. The addition of a suitable crosslinker changes a hydrogel’s structure, while improving its mechanical properties. The inclusion of a naturally-occurring crosslinker is of interest due to its inherent biocompatibility and reduced cytotoxicity [3]. One such compound which has attracted increased research activity over recent years is genipin (Figure 1), a naturally-occurring iridoid which can be extracted from the fruit of *Gardenia jasminoides* Ellis, an evergreen bush which is found in India, China, Japan, Vietnam, and South America [4,5,6]. Genipin is a bicyclic aglycone which contains a dihydropyran ring and an ester group. Its pure physical form is a clear, monoclinic, crystalline needle-like solid [7]. The key driving force behind investigating genipin’s crosslinking behaviour is that it is 5000 to 10,000 times less cytotoxic than the most common crosslinking reagent, glutaraldehyde [8]. Traditionally, genipin was used to treat pyrogenic infections, hematemesis, febrile diseases, and also as an external treatment for sprains and swellings [9]. Genipin derivatives, produced via the reaction of genipin with primary amine groups in the presence of oxygen, are highly stable when exposed to heat, pH, and light, and are used as a blue food pigment named “gardenia blue” [10].

The mechanism of the crosslinking reaction of genipin with chitosan has been studied over the past two decades. It has been reported that pH is the governing environmental factor affecting the reaction pathway [11,12]. The reaction mechanism that takes place under acidic conditions is described by several reaction mechanisms. The first mechanism involves an S_N_2 nucleophilic substitution reaction by which the ester group on the genipin molecule is replaced by a secondary amide linkage to a molecule of chitosan [13]. The second crosslinking reaction mechanism involves a nucleophilic attack of the C3 carbon atom by a primary amine group from a second molecule of chitosan forming an intermediate aldehyde group. The dihydropyran ring is then opened, resulting in another aldehyde group, which is then attacked by the secondary amine formed in the first step of the reaction to form a heterocyclic amide linkage [13]. In this way one molecule of genipin forms a single bifunctional crosslink between two molecules of chitosan [14]. Under more basic conditions, genipin molecules have been found to self-polymerise due to the nucleophilic attack by OH^−^ ions, allowing aldol condensation reactions to occur. This was reported to occur prior to crosslinking reactions with chitosan to form long bridges of genipin between chitosan chains [11]. These genipin oligomer bridges are terminated by a Schiff-base reaction with the amino groups on chitosan to form crosslinked networks with larger distances between chitosan chains [15].

The potential applications of genipin crosslinked hydrogels are vast, particularly when low cytotoxicity of the crosslinking agent is crucial. While currently using genipin at larger scales is limited due to cost (£290 per 125 mg, Sigma Aldrich, Gillingham, UK), the cost is likely to be reduced as the technology employed in genipin extraction is improved. Some identified potential applications of genipin with chitosan are in the preparation of elastic cartilage substitutes, the construction of carriers for the controlled release of drugs, the encapsulation of biological products and living cells, the biofabrication of synthetic tissues, such as muscle and arterial walls, and the dressing of wounds in animals and humans [16]. Viscoelastic properties of genipin-crosslinked chitosan hydrogels have been investigated previously. Moura et al. noted that the addition of genipin as a crosslinking agent reduces the gelation time compared to a chitosan-only solution and improves the elasticity and three-dimensional stability of the gel. This study also showed that the viscoelastic features of chitosan hydrogels can be “tuned” by changing the genipin loading and that the in situ formulation of the hydrogel made promising matrices for the encapsulation of cells and bioactive materials [17].

Crosslinking density within genipin-chitosan hydrogels has been found to have a marked impact on the macroporous structure and mechanical stiffness. Increasing the crosslinking density was found to improve the hydrogel’s cell bioadhesion properties and viability [18]. No undesired cytotoxic effects were detected. Electrospun nanofibrous chitosan scaffolds crosslinked by genipin are considered promising for both bone and cartilage tissue engineering [16]. Genipin crosslinked tissue scaffolds containing osteoblasts for the regeneration of bone tissue have shown progressive adhesion, proliferation and colonisation in vitro [19]. More recent endeavours have included seeding genipin-crosslinked hydrogels with stem cells in order to stimulate tissue regeneration [20].

Now that genipin has been shown to meet and outperform other more cytotoxic crosslinking agents, developing non-invasive methods to follow the gelation process and track changes in the crosslinking rate and crosslinking density becomes important. In this study, a non-invasive in situ method is based on genipin’s ability to fluoresce upon crosslinking. The advantages of fluorescence imaging are that it exhibits superior sensitivity, has low radiation energy, and can identify multiple independent biomarker reporters simultaneously [21]. There are many naturally-occurring molecules which exhibit intrinsic fluorescence, the most notable being the green fluorescent protein (GFP) that originates from the bioluminescent jellyfish *Aequorea Victoria.* GFP is used extensively as a reporter gene in monitoring gene expression and protein localisation in living cells as it does not require a substrate or interfere with cell growth or function [22]. The discovery and refinement of GFP was the subject of a Nobel Prize award in 2008, signifying the importance of reporter genes and fluorescence imaging in genetics and other imaging applications.

While research has been carried out into the genipin-crosslinked hydrogels’ reaction mechanisms [13,14,15], post-synthesis treatment [23], composition, and mechanical properties [24], a smaller number of papers are reporting on its fluorescent behaviour. Hurst and Novakovic investigated the fluorescence of genipin crosslinked chitosan—PVP hydrogels during post-synthesis swelling over a short period of time [25]. Chen et al. studied the fluorescence characteristics of the chitosan-genipin reaction in chitosan-coated alginate microcapsules which had been immersed in genipin solutions [26]. Hwang et al. [27] and Sundararaghavan et al. [28] investigated the fluorescence intensity whilst genipin crosslinking occurred for collagen-based hydrogels. The study presented here uses fluorescence intensity measurements as a non-invasive in situ method to follow sol-gel transition of genipin crosslinked chitosan-PVP hydrogels at 24 and 37 °C, using solutions prepared in deionised water, pH 4, and pH 10 buffers. In addition to presenting the average fluorescence values for each time point, three-dimensional representations of the fluorescence present in a hydrogel sample have also been produced from the data to observe how fluorescence varies over time at the surface of the hydrogel.

## 2. Materials and Methods

### 2.1. Materials

Genipin (≥98%, product code G4796), chitosan (medium molecular weight of 190,000–300,000 g/mol, 80% deacetylated, product code 448877), PVP (approximate molecular weight 40,000 g/mol, product code PVP40), and glacial acetic acid (>96%, product code ARK2183) were purchased from Sigma Aldrich (Gillingham, UK). Phthalate (pH 4) and borate (pH 10) buffers were purchased from Fisher Scientific (Loughborough, UK). All chemicals were used as received.

### 2.2. Preparation of Hydrogels

A 1.5% (*w*/*v*) solution of chitosan was prepared by dissolving chitosan in a deionised aqueous solution of 1% (*v*/*v*) acetic acid. This was stirred at room temperature for 24 h in a sealed glass vessel using a magnetic stirrer and created a pale yellow viscous solution. A 5% (*w*/*v*) solution of PVP was prepared by dissolving PVP in deionised water. This was then stirred at room temperature for 24 h in a sealed glass vessel using a magnetic stirrer to create a transparent solution. A 0.5% (*w*/*v*) aqueous genipin solution was prepared by dissolving genipin in deionised water. To ensure that all of the genipin crystals had dissolved, the solution was partially submerged in an ultrasonic bath (QS12, Ultrawave, Cardiff, UK) for 30 min after initial degassing at room temperature to aid dissolution. Defined volumes of chitosan (0.15 mL) and PVP (0.75 mL) were pipetted into labelled 5 mL, 10 mm diameter glass vials with secure twist-close caps. These were then magnetically stirred for 10 min to ensure a homogeneous solution. Genipin (0.1 mL) was then pipetted into each glass vial and each solution was magnetically stirred for a further 5 min to ensure that the genipin had been uniformly distributed throughout each sample. Solutions were then transferred into a 24-well microplate (Vision plate 24, 4titude, Surrey, UK). To prepare the stock solutions at different pH values, chitosan and PVP solutions were created as above, but the deionised water was replaced by the phthalate or borate buffers. In all cases, genipin was dissolved in water as it was found that the change in pH was negligible.

### 2.3. Calculation of Molar Ratios of Components in the Hydrogels

Molar ratios were calculated following a published procedure [23]. As chitosan has a range of molecular weights (Section 2.1), maximum and minimum concentrations were found and average values were used (Table 1). Although not ideal, this compromise to accuracy is required when using naturally-sourced biopolymers. The weight by volume values were divided by the molecular weight and then divided again by the 100 mL to find the concentration of the stock solution in mol/mL as shown in Table 1. The molar concentrations of PVP and genipin were calculated in the same way but, as their molecular weight is exact, there was no need for averaging.

The concentrations found in Table 1 were then multiplied by the volume of the component which is being added to the hydrogel mixture and divided by the total volume of the mixture to give the initial molar concentration of each constituent in the hydrogel (Table 2). The total volume of the hydrogel mixture was approximately 1 mL. The molar ratios were then calculated by dividing each molar concentration by the average molar concentration of chitosan to obtain normalised values. A single hydrogel composition, defined in Table 2, was investigated.

### 2.4. Fluorescence Intensity Measurements

The fluorescence intensity of samples was measured using a UV-VIS spectrophotometer (BMG LabTech, FLUOstar Omega, BMG Labtech, Aylesbury, UK) in fluorescence intensity mode. A 24-well fluorescence microplate was used and the spectrophotometer was connected to a laptop with Omega software (BMG Labtech, Aylesbury, UK) installed. The total volume of each hydrogel sample in a single well was kept at approximately 1 mL to avoid cross-contamination between the wells in the microplate, as the microplate moved laterally within the UV-VIS spectrophotometer. The wavelengths used were 485 nm for the excitation wavelength filter and 520 nm for the emission wavelength filter. Fluorescence was imaged top-down.

**Scheme 1:**
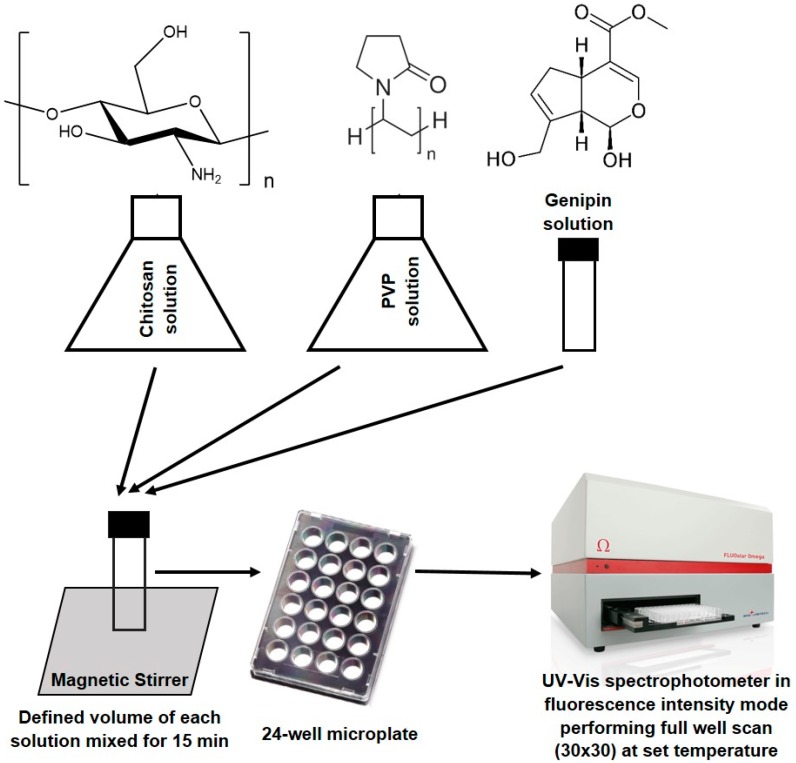
A schematic representation of the gel fabrication process.

The Omega software allows for two types of fluorescence intensity imaging: end point scans and well scans. In this study well scans were used as this method can provide a detailed representation of where within each well fluorescence is being detected. This also allows for the uniformity of any fluorophores present in samples to be observed over time as chemical crosslinking occurs. Although the software allows for a maximum density of a 30 × 30 matrix across the single well, 716 of these data points were actually measured due to the circular shape of the well. One scan per hour was performed for each sample over a 24 h period. Control samples of chitosan, PVP, genipin, and deionised water (1 mL) were also analysed. A schematic representation of the gel fabrication process is given in Scheme 1.

## 3. Results and Discussion

**Figure 2:**
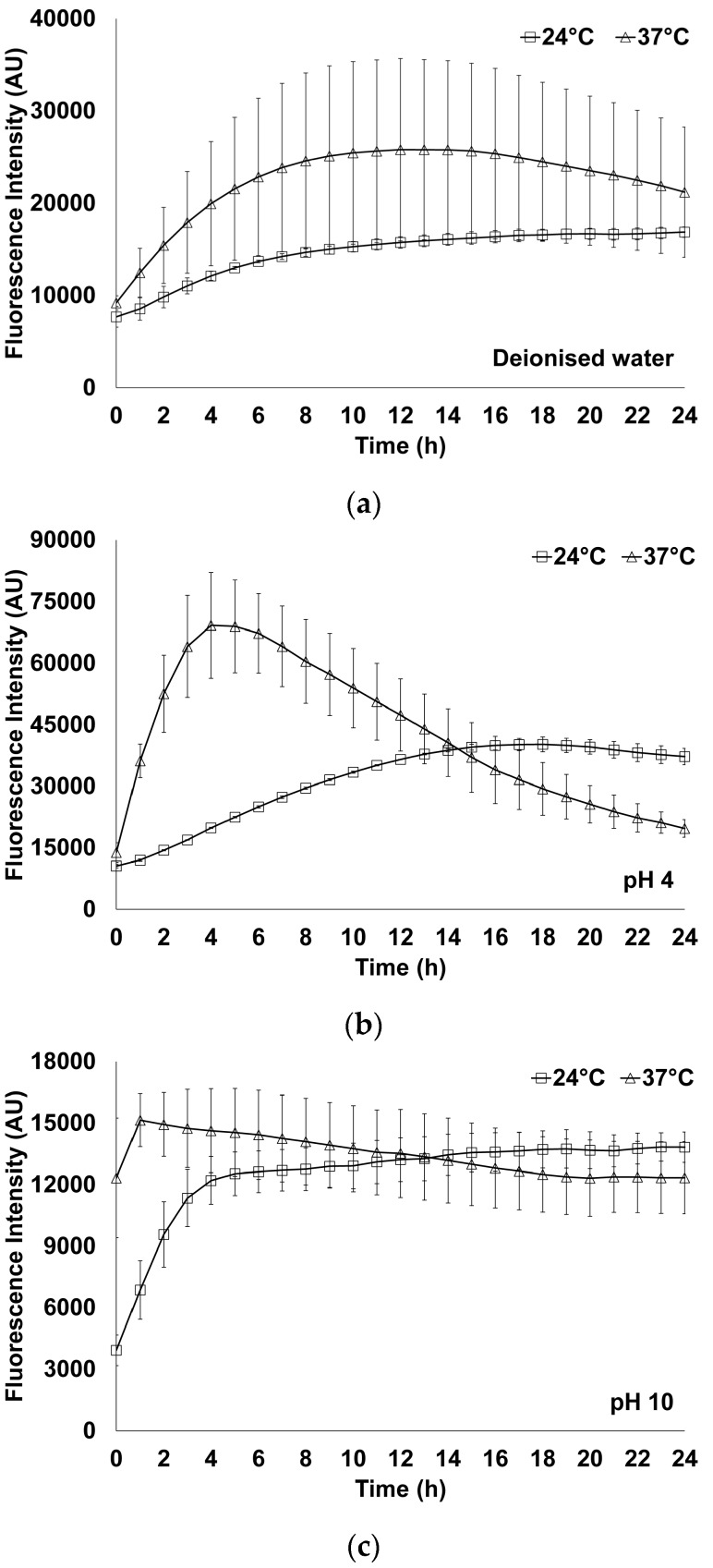
Fluorescence intensity of hydrogels during synthesis at 24 and 37 °C in (**a**) deionised water; (**b**) at pH 4; and (**c**) at pH 10.

All experiments were conducted two times, on different days, using identical settings for the measurements. Experiments were performed in a random order. The error bars on all of the results graphs are ±one standard deviation from the mean. Fluorescence intensity was imaged dynamically every hour for 24 h at 24 and 37 °C, starting measurements once the components were mixed together. Samples prepared using deionised water, pH 4 and pH 10 solutions were screened. Average values of fluorescence intensity for each well were calculated and the results are given in Figure 2.

From Figure 2 it can be noted that in all of the solutions studied fluorescence intensity initially increases with the increase in temperature. This behaviour is expected and follows a trend noted in the majority of chemical reactions, i.e., the rate of reaction increases as temperature increases. In all cases the deviation from the mean value is higher at higher temperature. This may be due to the evaporative loss of water and water condensation. In all samples studied at 37 °C, following the initial increase, fluorescence intensity decreases. This decrease indicates a decrease in the degree of crosslinking. A decrease in fluorescence intensity after a peak value was also found experimentally by Hwang et al. when genipin at concentrations of 10 mM was added to collagen-based hydrogels [27]. It may be caused by interaction of the hydrogel with methanol which is the main by-product formed. Based on the amount of genipin used in each 1 mL sample (2.21 × 10^−5^ mol), 0.7072 mg of methanol could be theoretically formed. As the reference dose for oral exposure to methanol is 2 mg/kg/day (https://www.epa.gov/iris) the suitability of these hydrogels for biomedical applications is unhindered. At the same time, it may be noted that higher concentrations of genipin can be used. Although less pronounced, a decrease in fluorescence intensity following the peak can also be observed at 24 °C for pH 4 hydrogels. Figure 2 also demonstrates the distinct effect pH has on fluorescence intensity. At both 24 and 37 °C, by moving from pH 4 to pH 10, a decrease in fluorescence intensity is noted. This observation supports the reaction mechanism postulated under more basic conditions. At pH 10, genipin molecules are suggested to self-polymerise prior to crosslinking reactions with chitosan, reducing the number of molecules available for reaction with the amino groups on chitosan [11]. Comparing the average fluorescence of the 24 h readings in pH 4 and pH 10 samples, it can be noted that the decrease in fluorescence is greater at 24 °C (63% drop) compared to 37 °C (35% drop). This suggests the interplay between competing processes which have different reaction rates: genipin crosslinking chitosan molecules and genipin oligomerising.

**Figure 3:**
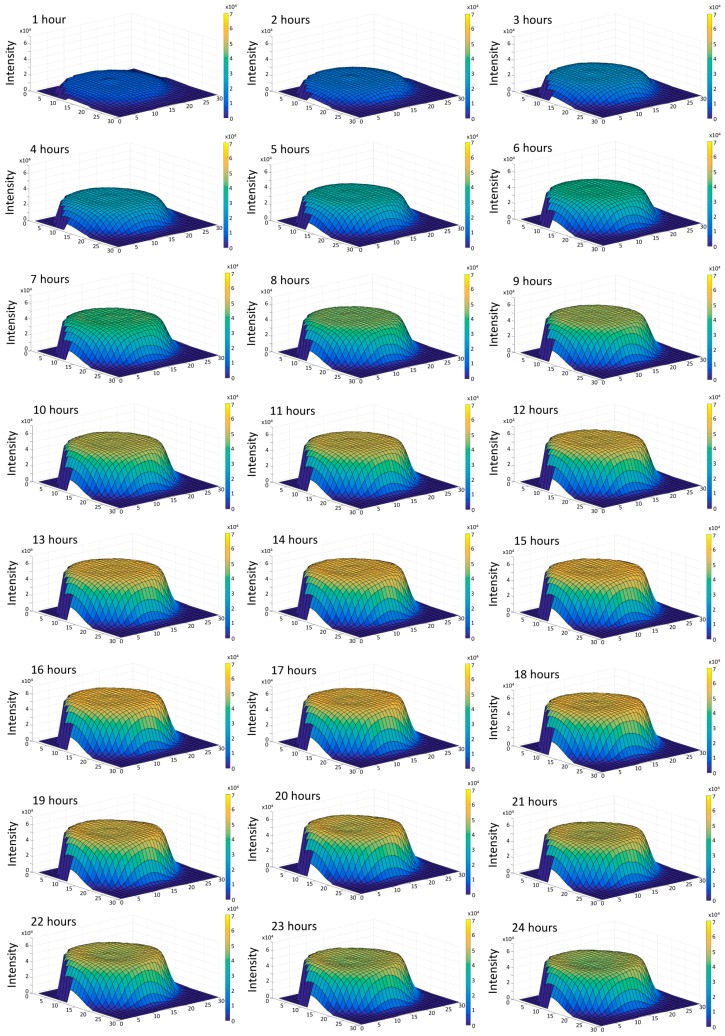
3D change in fluorescence intensity of hydrogel prepared in pH 4 buffer and screened at 24 °C, every hour, over a 24 h period.

To further look into the dynamic changes of fluorescence intensity, three-dimensional (3D) interpretations were created in MATLAB (MathWorks, Cambridge, UK) to show, physically, where the fluorophores were positioned within the hydrogel surface as they underwent crosslinking. While the fluorescence studies were conducted in more detail for all samples, the results for the hydrogel prepared in pH 4 and studied at 24 °C are shown in Figure 3 as an example. This particular hydrogel was chosen to further look into the subtle fluorescence intensity drop following the peak value (Figure 2b).

In Figure 3, the *x* and *y* axes represent the physical base of the well, whilst the *z* axis represents the fluorescence intensity in arbitrary units (AU). It may be noted that each surface is slightly off-centre. This is attributed to the motion of the microplate between readings. The fluorescence is observed to be highest in the centre of the sample. This is shown in the surface plots by a slight rise in fluorescence intensity in the centre of the raised surface. Two main 3D shapes were observed: “thimble”-shaped flat-topped domes and “concave thimble”-shaped domes. Relatively flat-topped “thimble”-shaped domes were associated with a gradual increase in fluorescence intensity i.e., an increase in crosslinking density, while “concave thimble”-shaped domes were related to fluorescence degradation, which is considered to relate to the degradation of the crosslinks formed. As previously mentioned, there is no fluorescence detected in the corners of each surface plot due to the wells in the microplate being circular. After twelve hours, the fluorescence intensity had risen at an even rate. The fluorescence intensity of the peak of the hydrogel is approaching twice that after six hours of crosslinking. This suggests that the hydrogel’s rate of crosslinking had been constant until this point in the experiment. The hydrogel’s fluorescence intensity peaks by 18 h. Six hours later (24 h well scan), the fluorescence intensity had decreased uniformly throughout the hydrogel sample by 8% suggesting some level of crosslink degradation. Fluorescence intensity throughout the surface is not uniform and, therefore, averaging fluorescence intensity is less informative.

It should be noted that it is difficult to standardise fluorescence measurements between different facilities as there are several factors which cause variance between measurements. Fluorescence is measured in arbitrary units (AU), and this will vary depending on the equipment used, the settings of the equipment, and the method of detection. As some fluorophores show fluorescence over a broad range of excitation wavelengths and different fluorophores fluoresce at different wavelengths entirely, this makes it challenging to compare measurements of gels which are not comprised of the same constituent chemicals. Therefore, fluorescence imaging cannot be used as a wider standardised comparison technique. However, for the purpose of comparing hydrogel samples which are made of the same compounds in a singular environment, fluorescence imaging is an appropriate method to compare the amount of crosslinking present in samples. Furthermore, as shown here, fluorescence imaging can provide an indication of the uniformity of samples.

## 4. Conclusions

Fluorescence intensity measurements were found feasible for detecting fluorophores which were the product of crosslinking functional groups (Figure 2 and Figure 3). Both temperature and pH have been found to affect sol-gel transition in the hydrogels studied. Importantly, in some mixtures, following an initial peak in fluorescence intensity, a decrease in intensity is observed, suggesting crosslink degradation. These observations were more pronounced at 37 °C than at 24 °C. The transition from acidic (pH 4) to basic (pH 10) solution resulted in reduced fluorescence intensity. This observation supports the postulated crosslinking mechanism [15] suggesting that under more basic conditions genipin molecules self-polymerise, reducing the number of molecules available for reaction with the amino groups on chitosan. As wells were scanned at maximum density every hour, a detailed representation of how fluorescence varied over time at the surface of the hydrogel samples was possible. The method discovered the non-uniform distribution of fluorophores present at the gel surface as chemical crosslinking occurred. This demonstrates that averaging fluorescence intensity measurements or measuring fluorescence in a limited area of the gel can give incomplete information. While the complex interaction between hydrogel components requires further studies to relate fluorescence intensity measurements with mechanical stability, measurement of fluorescence intensity is demonstrated to be a suitable method when genipin is employed as a crosslinking agent. The non-destructive nature of the method is of particular relevance to medical applications employing genipin-crosslinked biocompatible scaffolds, due to the potential for real-time monitoring of material formation, conformation change, and degradation.

## Figures and Tables

**Table 1:** Stock solution concentrations.

Components	*w*/*v* %	Solution (mL)	MW (g/mol)	mol/mL
Chitosan Low MW	1.5	100	190,000	7.89 × 10^−8^
Chitosan High MW	1.5	100	300,000	5.00 × 10^−8^
Chitosan Average MW	1.5	100	245,000	6.44 × 10^−8^
PVP	5	100	40,000	1.25 × 10^−6^
Genipin	0.5	100	226	2.21 × 10^−5^

**Table 2:** Molar concentrations of hydrogel constituents.

Components	Volume (mL)	mol/dm^3^	Molar ratio
Chitosan Low MW	0.15	1.18 × 10^−5^	-
Chitosan High MW	0.15	7.50 × 10^−6^	-
Chitosan Average	0.15	9.67 × 10^−6^	1
PVP	0.75	9.38 × 10^−4^	97
Genipin	0.1	2.21 × 10^−3^	229
